# Supplementary material for: Systematic imaging reveals features and changing localization of mRNAs in Drosophila development
Source: eLife. 2015 Apr 2;4:e05003. doi: 10.7554/eLife.05003 (PMC4384636; doi:10.7554/eLife.05003)
Supplement: Supplementary file 1. — Gene set definitions. Definition of Gene Sets used in this analysis from the ovary and the embryo (Lecuyer et al., 2007) FISH annotation matrices. DOI: http://dx.doi.org/10.7554/eLife.05003.019 [file elife05003s001.docx]

**Supplementary Table S1.** Gene Set definitions derived from the ovary or the embryo (Lecuyer et al., 2007) FISH annotation matrix.

| **Gene Set** | **Number of genes** | **Collapsed Annotation Terms** |
| --- | --- | --- |
| no signal | 2366 | no signal at all stages |
| ubiquitous | 3647 | ubiquitous signal at all stages |
| specific | 1290 | each gene with >1 annotation terms |
| Cellular (binary matrix) |  | stage1, stage2_to_7, stage8, stage9, stage10, germline cells ubiquitous (stage 1-10), oocyte (stage 1-10), nurse cells (stage 1-10), somatic cells (stage 1-10), follicle cells (stage 1-10), interfollicular stalk cells (stage 1-10), ubiquitous (stage 1-10), terminal filament, cap cells, escort cells, follicle stem cells, posterior follicle cells, germline stem cells, cystoblast, presumptive nurse cells, presumptive oocyte, anterior follicle cells (stage 2-8), border cells (stage 9-10), posterior follicle cells (stage 2-10), centripetally migrating follicle cells, follicle cells overlaying the oocyte, squamous follicle cells. |
| Nuclear (binary matrix) |  | nuclear foci in nurse cells (stage 1-10), nuclear foci in somatic cells (stage 1-10). |
| Subcellular (binary matrix) |  | anterior restriction (stage 2-10), posterior restriction (stage 2-10), perinuclear in nurse cells/somatic cells (stage 2-10), cytoplasmic foci (stage 1-10), apical restriction (stage 2-10), basal restriction (stage 2-10), cortical enrichment (stage 1-10), oocyte enrichment ubiquitous. |
| cellular (mutually exclusive gene set) | 309 | annotation for nurse cells, follicle cells and ovary that are not associated with subcellular specific terms (cellular TRUE, subcellular FALSE, nuclear FALSE). |
| subcellular (mutually exclusive gene set) | 790 | subcellular annotations in nurse cells, follicle cells and oocyte (cellular TRUE, subcellular TRUE, nuclear TRUE/FALSE). |
| nuclear (mutually exclusive gene set) | 191 | nuclear foci in nurse cells, follicle cells, oocyte nucleus (cellular TRUE, subcellular FALSE, nuclear TRUE). |
| oocyte-enriched | 591 | stage 2-7 oocyte enrichment |
| anterior | 106 | stage 8/9/10 oocyte anterior |
| Posterior | 119 | stage 8/9/10 oocyte posterior |
| perinuclear | 51 | stage 2-7/8/9/10 perinuclear |
| apical | 30 | stage 2-7/8/9/10 apical |
| basal | 9 | stage 2-7/8/9/10 basal |
| embryo subcellular localization | 1081 | stage 1-5 Subcellular localization patterns |
| embryo anterior | 14 | stage 1 Anterior localization, stage 2 Anterior localization |
| embryo posterior | 105 | stage 1 Posterior localization, Pole plasm, stage 2 Posterior localization |
| embryo basal | 126 | stage 2 Basal localization, stage 2 Basal enrichment |
| embryo apical | 187 | stage 2 Apical localization, stage 2 Apical enrichment, stage 2 Diffuse apical localization |
| embryo nuclear | 74 | stage 2 Accumulation entire nucleus, Intranuclear accumulation, Accumulation in subset nuclei, Accumulation nuclear subregion, Accumulation entire nucleus, Intranuclear accumulation, Accumulation in subset nuclei, Accumulation nuclear subregion |
| Microtubule minus ends in oocytes |  | stage 2-7 oocyte enrichment; stage 8 anterior; stage 9 anterior; stage 10 anterior. |
| Microtubule plus ends in oocytes |  | stage 8 posterior; stage 9 posterior; stage 10 posterior. |
